# Cytotoxic Psammaplysin Analogues from the Verongid Red Sea Sponge *Aplysinella* Species

**DOI:** 10.3390/biom9120841

**Published:** 2019-12-08

**Authors:** Lamiaa A. Shaala, Diaa T. A. Youssef

**Affiliations:** 1 Natural Products Unit, King Fahd Medical Research Center, King Abdulaziz University, Jeddah 21589, Saudi Arabia; 2 Department of Medical Laboratory Technology, Faculty of Applied Medical Sciences, King Abdulaziz University, Jeddah 21589, Saudi Arabia; 3 Suez Canal University Hospital, Suez Canal University, Ismailia 41522, Egypt; 4 Department of Natural Products, Faculty of Pharmacy, King Abdulaziz University, Jeddah 21589, Saudi Arabia; 5 Department of Pharmacognosy, Faculty of Pharmacy, Suez Canal University, Ismailia 41522, Egypt

**Keywords:** Order Verongiida, Red Sea sponges, *Aplysinella* species, bromotyrosine-derived metabolites, psammaplysin A, psammaplysin E, psammaplysin Z and 19-hydroxypsammaplysin Z, cytotoxicity, human cancer cell lines

## Abstract

As part of our ongoing interest to identify bioactive chemical entities from marine invertebrates, the Red Sea specimen of the Verongid sponge *Aplysinella* species was studied. Repeated chromatographic fractionation of the methanolic extract of the sponge and HPLC purification of the cytotoxic fractions led to the isolation and the identification of two new compounds, psammaplysin Z and 19-hydroxypsammaplysin Z (**1** and **2**), together with the previously reported psammaplysins A (**3**) and E (**4**). The structural determination of **1**–**4** was supported by interpretation of their NMR and high-resolution mass spectra. Psammaplysins A and E displayed cytotoxic activity against MBA-MB-231 and HeLa cell lines with IC_50_ values down to 0.29 µM. On the other hand, psammaplysin Z and 19-hydroxypsammaplysin Z were moderately cytotoxic, indicating the importance of the terminal amine and 2-(methylene)cyclopent-4-ene-1,3-dione moieties in **3** and **4** for potent cytotoxic activity.

## 1. Introduction

**Figure 1:**
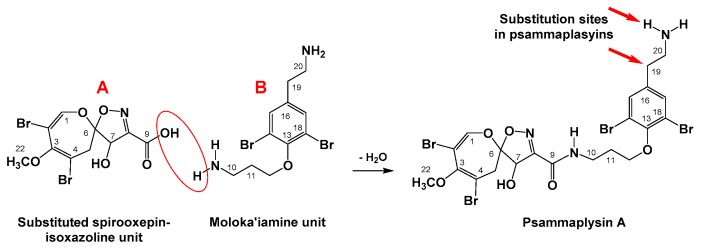
Subunits of the psammaplysins’ backbone.

Marine invertebrates are considered as an excellent source of biologically active biomolecules. Marine sponges, phylum Porifera, represent an attractive subject for chemists and pharmacologists, who target marine-derived biomolecules. Members of the order Verongiida are characterized by production of brominated compounds that are biosynthesized from bromotyrosine [1]. Compounds possessing the rare dibrominated 1,6-dioxa-2-azaspiro[4.6]undeca-2,7,9-triene moiety (spirooxepinisoxazoline) are derived from bromotyrosine and are named psammaplysins [2,3,4,5,6,7,8,9,10], ceratinamides [9,11] and ceratinadins [12]. Compounds with the spirooxepinisoxazoline moiety were reported mainly from members of the Verongiida [2,3,4,5,6,7,8,9,10,12] with only two representatives from the order Dictyoceratida [11,13]. Psammaplysins’ backbone consists of two dibrominated subunits, 8,10-dibromo-4-hydroxy-9-methoxy-1,6-dioxa-2-azaspiro[4.6]undeca-2,7,9-triene-3-carboxylic acid (subunit A) and 3-(4-(2-aminoethyl)-2,6-dibromophenoxy)propan-1-amine subunit (subunit B, moloka’iamine) [14], connected together through an amidic linkage between the carboxylic moiety (C-9) of the substituted spirooxepinisoxazoline unit and the terminal amino group at C-10 of the moloka’iamine (Figure 1) to give the first reported compound of this class, psammaplysin A, (*N-*(3-(4-(2-aminoethyl)-2,6-dibromophenoxy)propyl)-8,10-dibromo-4-hydroxy-9-methoxy-1,6-dioxa-2-azaspiro[4.6]undeca-2,7,9-triene-3-carboxamide) [2]. Interestingly, moloka’iamine (subunit B) and its substituted derivatives were reported from several Verongid sponges [9,13,14,15], but there is no single report in the literature about the existence or isolation of the separated dibrominated spirooxepinisoxazoline moiety (subunit A). The substituted and dibrominated spirooxepinisoxazoline unit has been always associated with the moloka’iamine moiety via an amidic linkage [2,3,4,5,6,7,8,9,10,11,12,13]. This can be explained by the necessity of such a combination as a defense tool for sponges against predators in the field.

To date, 41 bromotyrosine derivatives with the spirooxepinisoxazoline skeleton have been isolated. These compounds include psammaplysins A−Y (25 compounds), 19-hydroxy derivatives of psammaplysins E, P, Q, S, T, U, W, and X (eight compounds), psammaplysin K-dimethyl acetal (one compound) [2,3,4,5,6,7,8,9,10,11], ceratinamides A and B and 19-hydroxyceratinamide A (three compounds) [9,10], ceratinadins E and F (two compounds) [12], and recently, frondoplysins A and B (two compounds) [13]. Psammaplysins were reported mainly from two orders including several representatives from the genera *Aplysinella, Psammaplysinella*, *Pseudoceratina*, and *Suberea* of the order Verongida [2,3,4,5,6,7,8,9,10,12] and only two representatives, *Hyatella* sp. [11] and *Dysidea frondosa* [13], from the order Dictyoceratida. The common substitution patterns on the psammaplysins’ backbone exist only on the terminal ethylamine (CH_2_CH_2_NH_2_) part of the moloka’iamine subunit. Hydyoxylation or acylation at C-19 and/or *N*-alkylation or acylation with different fatty acids’ moieties represent the major substitution patterns on the psammaplysins’ skeleton [2,3,4,5,6,7,8,9,10,11]. Exceptionally, ceratinadins E and F possess two or three moloka’iamine subunits attached to the spirooxepinisoxazoline unit through amidic linkages [12]. Interestingly, frondoplysins A and B from *Dysidea frondosa* [13] possess an unusual meroterpene unit attached to the terminal amine of the psammaplysins [13].

As a part of our ongoing work on the Red Sea Verongiid sponges [15], we investigated the cytotoxic extracts of the sponge *Aplysinella* species. Two new bromotyrosine-derived compounds, psammaplysin Z (**1**) and 19-hydroxypsammaplysin Z (**2**), together with the known psammaplysins A (**3**) [2] and E (**4**) [4], were isolated. The structural determination of **1**–**4** was established by assignment of their NMR and high-resolution electrospray ionization mass spectrometry (HRESIMS) data. Herein, the assignment of the structures as well as the cytotoxic activities of **1**–**4** was reported.

## 2. Results and Discussion

### 2.1. Isolation of Compounds ***1***–***4***

**Figure 2:**
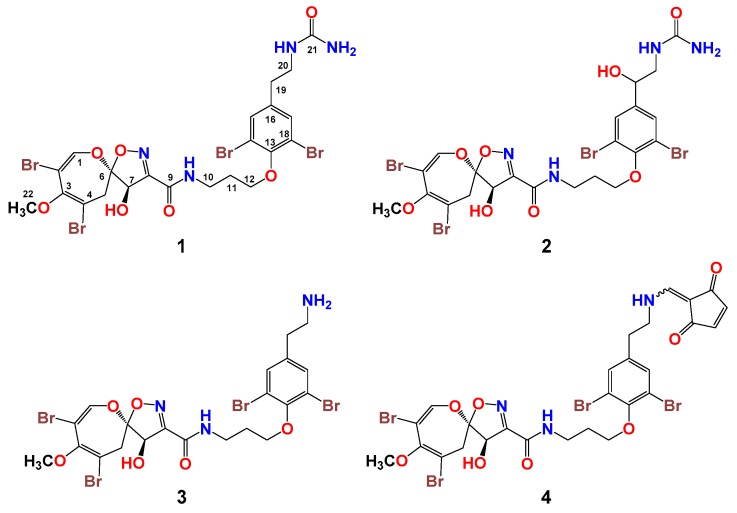
Chemical structures of psammaplysin Z (**1**), 19-hydroxypsammaplysin Z (**2**), psammaplysins A (**3**) and E (**4**).

Extraction of the sponge *Aplysinella* species with MeOH and successive partition of the aqueous MeOH extract against hexane, CH_2_Cl_2_, and EtOAc afforded three organic fractions. The cytotoxic CH_2_Cl_2_ fraction was acidified with dilute HCl and re-extracted with CH_2_Cl_2_. Repeated chromatographic fractionation of the organic extract, successive fractions on SiO_2_, Sephadex LH-20, and Sep-Pak C18 cartridge columns, and purification of the cytotoxic subfractions on a reversed-phase C18 HPLC column afforded compounds **1**–**4** (Figure 2).

### 2.2. Structural Determination of Compounds ***1***–***4***

**Figure 3:**
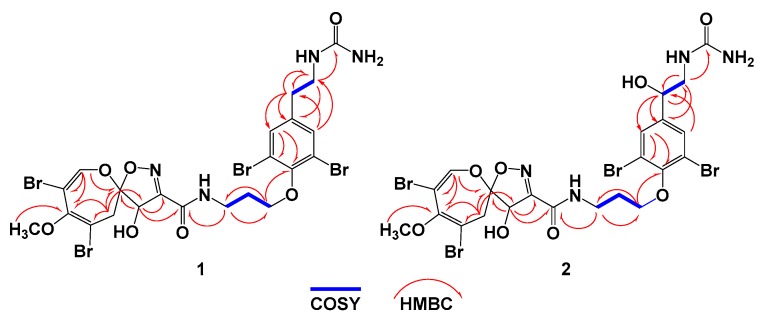
Observed COSY and ^2^*J*_CH_ and ^3^*J*_CH_ HMBC correlations of **1** and **2**.

Psammaplysin Z (**1**) (Figure 2) was isolated as an optically active yellow solid with the molecular formula C_22_H_24_Br_4_N_4_O_7_, requiring 11 degrees of unsaturation. The tetrabrominated nature of **1** was supported from the 1:4:6:4:1 ion cluster, which was displayed by the pseudomolecular ion peaks in the HRESIMS (Supplementary Figure S1) at *m*/*z* values of 772.8, 774.8, 776.8, 778.8, and 780.8 [M + H]^+^. Investigation of the NMR spectra of **1** including ^1^H (Supplementary Figure S2), ^13^C (Supplementary Figure S3), DEPT (Supplementary Figure S4), ^1^H-^1^H COSY (Supplementary Figure S5), and HSQC (Supplementary Figure S6) experiments supported the presence of four methine groups, six methylenes group, one methyl group, and 11 quaternary carbons. The signals at δ_H/_**_C_** values of 7.16 (1H, s)/146.9 (CH, C-1), 104.5 (qC, C-2), 150.0 (qC, C-3), 104.6 (qC, C-4), 3.38 (1H, d, *J* = 16.0 Hz) and 3.08 (1H, d, *J* = 16.0 Hz)/38.4 (CH_2_, C-5), 121.0 (qC, C-6), 5.00 (1H, s)/80.5 (CH, C-7), 159.0 (qC, C-8), 160.8 (qC, C-9), and 3.67 (3H, s)/59.4 (CH_3_, C-22) are characteristic for the 2,3,4,7,9-penta-substituted spirooxepinisoxazoline unit [2,3,4,5,6,7,8,9,10,11,12,13]. The HMBC experiment (Supplementary Figure S7) supported and secured the placement of the substituents on the spirooxepinisoxazoline moiety as “2,4-dibromo-7-hydroxy-3-methoxy-9-carbonyl” (Figure 2). For example, the HMBC correlations from H-1 to C-2, C-3, and C-6, from H_2_-5 to C-3, C-4, and C-6, from H-7 to C-6, C-8, and C-9, and from OCH_3_ to C-3 (Table 1 and Figure 3) supported the quaternary carbons’ assignment, the placement of the substituents on the spirooxepinisoxazoline skeleton, and the assignment of the first subunit of **1**.

The second substructure of **1** was assigned as the *N,N*-disubstituted moloka’iamine moiety, as supported by investigation of the ^1^H, ^13^C, COSY, and HSQC experiments. In the second substructure of **2**, two spin–spin coupling systems with vicinal couplings (*^3^J*_HH_ = 6.5–6.6 Hz) were traced from H_2_-10 to H_2_-12 and between H_2_-19 and H_2_-20, respectively. The δ_H/_**_C_** values of 3.64 (2H, t)/38.1 (CH_2_, C-10), 2.15 (2H, quin.)/30.7 (CH_2_, C-11), 4.08 (2H, t)/72.2 (CH_2_, C-12), 153.0 (qC, C-13), 119.1 (2 × qC, C-14, C-18), 7.49 (2H, s)/134.4 (2 × CH, C-15, C-17), 139.8 (qC, C-16), 2.78 (2H, t)/35.1 (CH_2_, C-19), and 3.46 (2H, t)/40.1 (CH_2_, C-20) supported the presence of the *N,N*-disubstituted moloka’iamine moiety [14,15]. The HMBC correlations (Table 1 and Figure 3) from H_2_-10 to C-11 and C-12, from H_2_-11 to C-10 and C-12, from H_2_-12 to C-10, C-11 and C-13 (δ**_C_** = 153.0), from H-15/17 to C-13, C-14/C-18 (δ**_C_** 119.1), and C-19 (δ**_C_** = 35.1), from H_2_-19 to C-16 (δ**_C_** = 139.8), C-17, and C-20, and finally from H_2_-20 to C-16 and C-19 supported the assignment of the second subunit of compound **1**.

The connection between the subunits of **1** via an amidic linkage (C-9) was supported by the HMBC correlations from H-7 (δ_H_ = 5.00) to C-9 (δ**_C_** = 160.8) and from H_2_-10 (δ_H_ = 3.64) to C-9 (δ**_C_** = 160.8) (Table 1 and Figure 3). This connection supported the basic structure of **1** with elements of C_21_H_22_Br_4_N_3_O_6_, which were counted for 10 degrees of unsaturation. The remaining elements of CONH_2_ were accounted for the last degree of unsaturation in **1** and were assigned as a terminal urea moiety. The attachment of the terminal urea moiety to the ethylamine of **1** was supported by the correlation in the HMBC experiment from H_2_-20 (δ_H_ = 3.46) to C-21 (δ**_C_** = 161.6) (Table 1 and Figure 3) to complete the planer structure of **1**. Accordingly, compound **1** was assigned as psammaplysin Z.

19-hydroxypsammaplysin Z (**2**) (Figure 2) was isolated as an optically active solid. Its HRESIMS spectrum displayed the molecular formula of C_22_H_24_Br_4_N_4_O_8_. The existence of four bromine atoms in **2** was established from the 1:4:6:4:1 pseudomolecular ion cluster in the HRESIMS (Supplementary Figure S8) at *m*/*z* values of 788.8, 790.8, 792.8, 794.8, and 796.8 [M + H]^+^. The molecular mass of **2** was 16 mass units lager than that of **1**, which suggested an additional oxygen atom in **2**. The extensive study and the assignment of the ^1^H (Supplementary Figure S9) and ^13^C NMR spectra (Supplementary Figure S10) and the DEPT (Supplementary Figure S11), ^1^H-^1^H COSY (Supplementary Figure S12), HSQC (Supplementary Figure S13), and HMBC experiments (Supplementary Figure S14) of **2** (Table 2) revealed similar NMR signals to those of psammaplysin Z (**1**) (Table 1).

The difference between the NMR spectra of compounds **1** and **2** was related to the signals of the ethylamine part of the moloka’iamine moiety (Figure 2 and Table 1 and Table 2). The NMR signals of the ethylamine fragment (C-19 and C-20) at δ_H/C_ values of 2.78 (2H, t)/35.1 (CH_2_, C-19) and 3.46 (2H, t)/40.1 (CH_2_, C-20) (Table 1) were absent in **2**. Instead, new signals at δ_H/C_ values of 4.69 (1H, dd)/71.5 (CH, C-19), 3.47 (1H, dd), and 3.31 (1H, m)/46.2 (CH_2_, C-20) were observed in **2** (Table 2), suggesting the hydroxylation of C-19. This was supported from downfield-shifted signals of H-19/C-19 at the δ_H/C_ value of 4.69/71.5 and the vicinal couplings between H-19 (δ_H_ = 4.69; *J* = 9.3 and 3.0 Hz) and H-20a (δ_H_ = 3.47) and H-20b (δ_H_ = 3.31) in the COSY experiment. Furthermore, the presence of the C-19 OH group was supported again by HMBC experiments. The correlations from H-19 (δ_H_ = 4.69) to C-15/C-17 (δ_C_ = 131.7), C-16 (δ_C_ = 143.2), and C-20 (δ_C_ = 46.2) and from H_2_-20 (δ_H_ 3.47 and 3.31) to C-16, C-15/C-17, C-19 (δ_C_ = 71.5), and C-21 (δ_C_ = 161.2) supported the hydroxylation of C-19 and completed the assignment of compound **2**. Accordingly, compound **2** was identified as 19-hydroxypsammaplysin Z.

Compounds **3** and **4** (Figure 2) were identified as the previously reported psammaplysin A [2] and psammaplysin E [4], respectively, after extensive and careful investigations of their 1D and 2D NMR and MS spectra and by comparison of their data with the literature [2,4].

Compounds **1**–**4** displayed optical rotations with negative signs and similar magnitudes. Therefore, it was more likely that psammaplysins **1**–**4** possessed the same biosynthetic pathway and shared similar absolute configurations of the spirooxepinisoxazoline moiety. In addition, the signs and the magnitudes of the optical rotations of compounds **1**–**4** were closely related to the literature [2,4,6,8,9,11]. Recently, the absolute stereochemistry of C-6 and C-7 of psammaplysin A was verified as 6*R* and 7*R*, respectively [16]. Therefore, we assume that compounds **1**–**4** share the same absolute configurations of C-6 and C-7 with psammaplysin A [16]. However, the stereochemistry of C-19 of 19 substituted psammaplysins [2,4,7,9,10,11] has not been established so far.

### 2.3. Biological Activities of Compounds ***1***–***4***

The reported pharmacological properties associated with the psammaplysins are diverse and include cytotoxicity, antimalarial, antiviral, antifouling, antimicrobial, and antioxidant activities. Psammaplysins A–C displayed cytotoxic activity against HCT 116 cell lines [3]. On the other hand, psammaplysin D possessed antiviral activity against HIV-1 virus at 0.1 µg/mL [4]. Psammaplysin E was cytotoxic to LoVo and KB cell lines at 5.0 µg/mL and showed moderate immunosuppressive activity [4]. It was also effective as a potent antimigratory agent against HeLa and MDA-MB-231 cells with IC_50_ values of 2.19 and 0.31 μM, respectively [15]. 19-hydroxypsammaplysin E displayed moderate antimalarial activity against 3D7 drug-sensitive strain of *P. falciparum* with an IC_50_ value of 6.4 μM [8]. Furthermore, psammaplysin F inhibited 80% of four bacterial strains at 50 µM [17] and showed antiplasmodial activity against 3D7 and Dd2 strains of *P. falciparum* with IC_50_ values of 0.87 and 1.4 μM, respectively [11]. When tested against the drug-resistant (K1) and drug-sensitive (FCR3) strains of *P. falciparum*, psammaplysin F was active with IC_50_ values of 3.77 and 2.45 µg/mL against these strains [12]. Recently, psammaplysin F was found to regulate the synthesis of stress granules, leading to increasing the efficacy of bortezomib and sorafenib [18]. Psammaplysin G showed 98% inhibition of the Dd2 cell strain of *P. falciparum* at 40 μM [11]. Similarly, psammaplysin H possessed potent antiplasmodial activity against the 3D7 strain with an IC_50_ value of 0.41 µM [6]. Psammaplysin H was also selective towards the 3D7 strain with a selectivity index (SI) of >97% [6]. Psammaplysins X and Y and 19-hydroxypsammaplysin X showed potent cytotoxicity towards six cancer cell lines with a GI_50_ value down to 0.8 µM [9]. When evaluated for their antiplasmodial activities, ceratinadin E displayed higher activity against FCR3 (IC_50_ = 0.77 μg/mL) and K1 (IC_50_ = 1.03 μg/mL) strains of *P. falciparum* than ceratinadin F, which displayed weak activity with an IC_50_ value of >12.5 μg/mL against the drug-resistant K1 strain [12]. Frondoplysin A potently inhibited the protein tyrosine phosphatase 1B with an IC_50_ value of 0.39 μM and displayed antioxidant activity in transgenic zebrafish without any cytotoxicity [13]. Finally, ceratinamides A and B displayed antifouling activity through inhibition of metamorphosis and settlement of the barnacle *B. amphitrite* with an ED_50_ range of 0.1–8.0 µg/mL [11].

In order to evaluate the cytotoxic activities of **1**–**4**, the compounds were screened in an MTT assay [19,20] against three human cancer cell lines, including the triple-negative breast cancer (MDA-MB-231, ATCC HTB-26), cervical carcinoma (HeLa, ATCC CCL-2), and colorectal carcinoma (HCT 116, ATCC CCL-247). Psammaplysin E (**4**) displayed the strongest activity againt MDA-MB-231 and HeLa cells with IC_50_ values of 0.29 and 2.1 µM, respectively, while psammaplysin A (**3**) was less active than **4** with IC_50_ values of 3.9 and 8.5 µM, respectively (Table 3). Moreover, both compounds were less active againt HCT 116 with IC_50_ values of 5.1 and 3.7 µM, respectively. On the other hand, compounds **1** and **2** were weakly active against MDA-MB-231 and HeLa cells with IC_50_ values ranging from 13.2 to 22.2 µM (Table 3), but when tested againt HCT 116, they showed better sensitivity towards this cell line with IC_50_ values of 8.2 and 7.0 µM, respectively.

The above data suggested, in general, the importance of the spirooxepinisoxazoline moiety in compounds **1**–**4** for cytotoxic activities. Additionally, the potency of the activities of the psammapysins depended mainly on the type of the substituents on the terminal amino group of the psammaplysins. For example, the presence of the 2-(methylene)cyclopent-4-ene-1,3-dione moiety in **4** caused higher and better sensitivity towards MDA-MB-231 and HeLa cells over the terminal amine in **3**, which was displayed by the potent cytotoxic activity of **4** against both cell lines down to 0.20 µM. Moreover, both **1** and **2** were weakly active against these cells as a result of the presence of the terminal urea moiety in both compounds. In addition, compounds **3** and **4** displayed better sensitity against HCT 116 than MDA-MB-231 and HeLa cell lines.

These results clearly indicated that both MDA-MB-231 and HeLa cells possess high sensitivity towards psammaplysins A (**3**) and E (**4**). Thus, psammaplysins A and E are considered as an attractive molecular scaffold for the development of anticancer drugs.

## 3. Material and Methods

### 3.1. General Experimental Procedures

The optical rotations of the compounds were acquired on a digital DIP-370 polarimeter (JASCO, Oklahoma City, OK, USA). The UV spectra were measured on a Hitachi 300 spectrometer (Hitachi High-Technologies Corporation, Kyoto, Japan). 1D and 2D NMR spectra were recorded on a Bruker Avance DRX 600 MHz (Bruker, Rheinstetten, Germany) spectrometer using CD_3_OD as a solvent. NMR spectra were referenced to the residual protonated solvent signal (CH_3_OH: 3.30 for ^1^H and 49.0 ppm for ^13^C). Positive-ion HRESIMS data were obtained with a Micromass Q-ToF spectrometer (Waters Corporation, Milford, MA, USA).

### 3.2. Biological Materials

The sponge, *Aplysinella* species, was harvested from the Red Sea coast off Jizan, KSA, at a depth of 15 m using SCUBA (Self-Contained Underwater Breathing Apparatus) diving. The sponge formed an irregular, thick, fleshy, and compressible yellow sheet with a thickness up to 7 cm thick. The color in life was bright yellow and turned into greenish-black after exposure to air and to completely black after storage in a 70% EtOH solution. The sponge was identified at the Department of Marine Sciences at the Faculty of Science of Suez Canal University (Ismailia, Egypt). A voucher sample of the sponge was stored at the Department of Pharmacognosy of Suez Canal University (Ismailia, Egypt) under collection #DY-78.

### 3.3. Purification of Compounds ***1***–***4***

The sponge was lyophilized, and the dried materials (520 g) were macerated in MeOH at room temperature for 24 h (3 × 2000 mL). The methanolic extracts were filtered and concentrated under vacuum to yield 7.3 g. The dried extract was suspended in a MeOH–H_2_O (6:4, *v*/*v*) solution and successively partitioned against hexane, CH_2_Cl_2_, and EtOAc. The cytotoxic CH_2_Cl_2_ extract (2.9 g) was dissolved in MeOH and diluted with aqueous HCl (pH: 3–4) followed by extraction with CH_2_Cl_2_. The concentration of the CH_2_Cl_2_ extract gave a dark yellow residue (1.20 g). The residue was partitioned over a SiO_2_ (Merck, 70–230 mesh ASTM) Vacuum Liquid Chromatography (VLC) column using hexane/EtOAc/MeOH with increasing polarity to give seven major fractions (A–G). Fractionation of the cytotoxic fraction (Fraction D) on a Sephadex LH 20 (0.25–0.1 mm, Pharmacia) column with MeOH gave four major subfractions (D1–D4). Fraction D2 (115 mg) was fractionated on a C-18 RP Sep-Pak cartridge (1.0 g, 6 cc vacuum cartridge, Waters) with a H_2_O/MeOH mixture (the volume percentage of MeOH in the mixture increased from 20% to 100% gradually) to give five subfractions (D2A–D2E). The cytotoxic fraction D2C (35 mg) was purified on an ODS HPLC column (250 × 10 mm, ARII, Cosmosil, Waters) using 75% MeOH to yield compounds **3** (7.3 mg) and **4** (3.7 mg). Similarly, fraction D3 (98 mg) was purified on a C-18 RP Sep-Pak cartridge (1.0 g, 6 cc vacuum cartridge, Waters) with a H_2_O/MeOH mixture (the volume percentage of MeOH in the mixture increased from 20% to 100% gradually) to give five subfractions (D3A–D3E). The cytotoxic fraction D3B (22 mg) was purified on an ODS HPLC column (250 × 10 mm, ARII, Cosmosil, Waters) using 80% MeOH to afford compounds **1** (2.3 mg), **2** (2.7 mg), and **4** (1.8 mg).

### 3.4. Spectral Data of the Compounds

Psammaplysin Z (**1**): pale yellow solid; [α]_d_: 54° (c 0.1, MeOH); UV (MeOH) λ_max_ (log ε): 304 (4.15) and 210 (4.85) nm; NMR data: Table 1; HRESIMS *m*/*z*: 772.8464 (calcd for C_22_H_25_Br_4_N_4_O_7_, [M + H]^+^, 772.8457).

19-hydroxypsammaplysin Z (**2**): yellow solid; [α]_d_: 61° (c 0.1, MeOH); UV (MeOH) λ_max_ (log ε): 305 (4.10) and 209 (4.90) nm; NMR data: Table 2; HRESIMS *m*/*z*: 788.8415 (calcd for C_22_H_25_Br_4_N_4_O_8_, [M + H]^+^, 788.8406).

### 3.5. Cytotoxicity Evaluation of Compounds ***1***–***4***

#### 3.5.1. Preparations of Cell Lines and Cell Culture

The cell lines used in this study were triple-negative breast cancer (MDA-MB-231 (ATCC HTB-26)), human cervical carcinoma (HeLa (ATCC CCL-2)), and colorectal carcinoma (HCT 116 (ATCC CCL-247)). The MDA-MB-231 cells were cultured in DMEM containing 10% FBS and 1% penicillin–streptomycin, while HeLa and HCT 116 cells were cultured in RPMI 1640 containing 10% FBS and 1% penicillin–streptomycin. All cells were cultured in an incubator at 37 °C with 95% humidity and 5% CO_2_.

#### 3.5.2. MTT Assay

The evaluation of the cytotoxicity of the compounds was carried out using an MTT assay as previously described [19,20]. In brief, the cells were incubated at 37 °C overnight in 5% CO_2_/air. Compounds **1**–**4** were added to the top row of a 96-well microtiter plate and downward diluted serially (1:4, *v*/*v*). The cells were incubated with the compounds for 72 h. Afterwards, viability of the cells was estimated at 490 nm using the Cell Titer 96 AQueous non-radioactive cell proliferation protocol (Promega, Madison, WI, USA) on a Molecular Devices Emax microplate reader. The IC_50_ values of the compounds (expressed in µM) were evaluated using the program SOFTmax PRO (Molecular Devices, Sunnyvale, CA, USA). DMSO and 5-fluorouracil (5-FU) were used as negative and positive controls, respectively. The IC_50_ values represent the average of three independent experiments. The data are presented in Table 3.

## 4. Conclusions

In conclusion, the fractionation of the cytotoxic fractions of the methanolic extract of the Red Sea sponge *Aplysinella* species gave two new psammaplysins’ derivatives, psammplysin Z (**1**) and 19-hydroxypsammaplyzin Z (**2**) together with the previously reported psammaplysins A (**3**) and E (**4**). Structures of the compounds were established by extensive interpretation of their NMR and MS spectroscopic data. In the cytotoxicity evaluation of the compounds, psammaplysin E (**4**) displayed the most potent activities against MDA-MB-231 and HeLa cells, while psammaplysin A (**3**), which contains a terminal amine, was less active than psammaplysin E. The results suggested the necessity of the rare 2-(methylene)cyclopent-4-ene-1,3-dione moiety in psammaplysin E for potent cytotoxic activity. On the other hand, psammpalysin Z (**1**) and 19-hydroxypsammaplysin Z (**2**) were moderately cytotoxic against both cell lines, which suggested again the preference of the free amino group in psammaplysin A (**3**) over the terminal urea moiety in **1** and **2** for the anticancer activity. Psammaplysins A and E showed potent cytotoxic activities against MDA-MB-231 and HeLa cells. Therefore, psammaplysins A and E, with the terminal amino group and 2-(methylene)cyclopent-4-ene-1,3-dione moiety, are considered as potential leads that can be developed as anticancer agents.

## Figures and Tables

**Table 1:** NMR spectral data of psammaplysin Z **(1**) (CD_3_OD).

Position	δ_C_ (mult.) *	δ_H_ (mult., *J* (Hz))	HMBC
1	146.9, CH	7.16 (1H, s)	C-2, C-3, C-6
2	104.5, qC		
3	150.0, qC		
4	104.6, qC		
5a 5b	38.4, CH_2_	3.38 (1H, d, 16.0) 3.08 (1H, d, 16.0)	C-3, C-4, C-6 C-3, C-4, C-6
6	121.0, qC		
7	80.5, CH	5.00 (1H, s)	C-6, C-8, C-9
8	159.0, qC		
9	160.8, qC		
10	38.1, CH_2_	3.64 (2H, t, 6.5)	C-9, C-11, C-12
11	30.7, CH_2_	2.15 (2H, quin., 6.5)	C-10, C-12
12	72.2, CH_2_	4.08 (2H, t, 6.5)	C-10, C-11, C-13
13	153.0, qC		
14	119.1, qC		
15	134.4, CH	7.49 (1H, s)	C-13, C-14, C-18, C-19
16	139.8, qC		
17	134.4, CH	7.49 (1H, s)	C-13, C-14, C-18, C-19
18	119.1, qC		
19	35.1, CH_2_	2.78 (2H, t, 6.6)	C-16, C-17, C-20
20	40.1, CH_2_	3.46 (2H, t, 6.6)	C-16, C-19, C-21
21	161.6, qC		
22	59.4, CH_3_	3.67 (3H, s)	C-3

* Signal multiplicities were determined from DEPT and HSQC experiments.

**Table 2:** NMR spectral data of 19-hydroxypsammaplysin Z (**2**) (CD_3_OD).

Position	δ_C_ (mult.) *	δ_H_ (mult., *J* (Hz))	HMBC
1	146.8, CH	7.14 (1H, s)	C-2, C-3, C-6
2	104.2, qC		
3	149.8, qC		
4	104.5, qC		
5a 5b	38.2, CH_2_	3.38 (1H, d, 16.0) 3.05 (1H, d, 16.0)	C-3, C-4, C-6 C-3, C-4, C-6
6	120.8, qC		
7	80.4, CH	4.97 (1H, s)	C-6, C-8, C-9
8	158.8, qC		
9	160.7, qC		
10	37.9, CH_2_	3.61 (2H, t, 6.5)	C-9, C-11, C-12
11	30.5, CH_2_	2.12 (2H, quin., 6.5)	C-10, C-12
12	72.0, CH_2_	4.06 (2H, t, 6.5)	C-10, C-11, C-13
13	153.5, qC		
14	119.2, qC		
15	131.7, CH	7.60 (1H, s)	C-13, C-14, C-18, C-19
16	143.2, qC		
17	131.7, CH	7.60 (1H, s)	C-13, C-14, C-18, C-19
18	119.2, qC		
19	71.5, CH	4.69 (1H, dd, 9.5, 3.0)	C-15, C-16, C-17, C-20
20a 20b	46.2, CH_2_	3.47 (1H, dd, 13.0, 3.0) 3.31 (1H, m)	C-16, C-19, C-21 C-16, C-19, C-21
21	161.2, qC		
22	59.3, CH_3_	3.63 (3H, s)	C-3

* Signal multiplicities were determined from DEPT and HSQC experiments.

**Table 3:** Cytotoxic activities of compounds **1**–**4**.

Compound	IC_50_ (μM)		
	MDA-MB-231	HeLa	HCT 116
1	19.4 ± 1.80	22.2 ± 2.0	8.2 ± 0.72
2	13.2 ± 0.45	17.6 ± 1.90	7.0 ± 0.65
3	3.90 ± 0.20	8.50 ± 0.81	5.1 ± 0.41
4	0.29 ± 0.05	2.10 ± 0.12	3.7 ± 0.31
5-fluorouracil (5-FU) *	13.0 ± 0.30	12.3 ± 0.25	4.6 ± 0.23

* Positive cytotoxicity control. Data are presented as the mean ± SD; *n* = 3.

## Supplementary Materials

Supplementary Materials are available at https://www.mdpi.com/2218-273X/9/12/841/s1. Figure S1: HRESIMS of psammaplysin Z (**1**), Figure S2: ^1^H NMR spectrum of psammaplysin Z (**1**) (CD_3_OD), Figure S3: ^13^C NMR spectrum of psammaplysin Z (**1**) (CD_3_OD), Figure S4: DEPT spectrum of psammaplysin Z (**1**) (CD_3_OD), Figure S5: ^1^H-^1^H COSY spectrum of psammaplysin Z (**1**) (CD_3_OD), Figure S6: HSQC spectrum of psammaplysin Z (**1**) (CD_3_OD), Figure S7: HMBC spectrum of psammaplysin Z (**1**) (CD_3_OD), Figure S8: HRESIMS of 19-hydroxypsammaplysin Z (**2**), Figure S9: ^1^H NMR spectrum of 19-hydroxypsammaplysin Z (**2**) (CD_3_OD), Figure S10: ^13^C NMR spectrum of 19-hydroxypsammaplysin Z (**2**) (CD_3_OD), Figure S11: DEPT spectrum of psammaplysin Z (**2**) (CD_3_OD), Figure S12: ^1^H-^1^H COSY spectrum of 19-hydroxypsammaplysin Z (**2**) (CD_3_OD), Figure S13: HSQC spectrum of 19-hydroxypsammaplysin Z (**2**) (CD_3_OD), Figure S14: HMBC spectrum of 19-hydroxypsammaplysin Z (**2**) (CD_3_OD).
