# Supplementary material for: Cytotoxic Psammaplysin Analogues from the Verongid Red Sea Sponge Aplysinella Species
Source: Biomolecules. 2019 Dec 8;9(12):841. doi: 10.3390/biom9120841 (PMC6995619; doi:10.3390/biom9120841)
Supplement: Supplementary file 1 [file biomolecules-09-00841-s001.pdf]

Supplementary Information

HRESIMS of psammaplysin Z (**1**) S1
^{1}H NMR spectrum of psammaplysin Z (**1**) (CD_3OD) S2
^{13}C NMR spectrum of psammaplysin Z (**1**) (CD_3OD) S3
DEPT spectrum of psammaplysin Z (**1**) (CD3OD) S4
^{1}H-^{1}H COSY spectrum of psammaplysin Z (**1**) (CD_3OD) S5
HSQC spectrum of psammaplysin Z (**1**) (CD_3OD) S6
HMBC spectrum of psammaplysin Z (**1**) (CD_3OD) S7
HRESIMS of 19-hydroxypsammaplysin Z (**2**) S8
^{1}H NMR spectrum of 19-hydroxypsammaplysin Z (**2**) (CD_3OD) S9
^{13}C NMR spectrum of 19-hydroxypsammaplysin Z (**2**) (CD_3OD) S10
DEPT spectrum of 19-hydroxypsammaplysin Z (**2**) (CD_3OD) S11
^{1}H-^{1}H COSY Spectrum of 19-hydroxypsammaplysin Z (**2**) (CD_3OD) S12
HSQC spectrum of 19-hydroxypsammaplysin Z (**2**) (CD_3OD) S13
HMBC spectrum of 19-hydroxypsammaplysin Z (**2**) (CD_3OD) S14

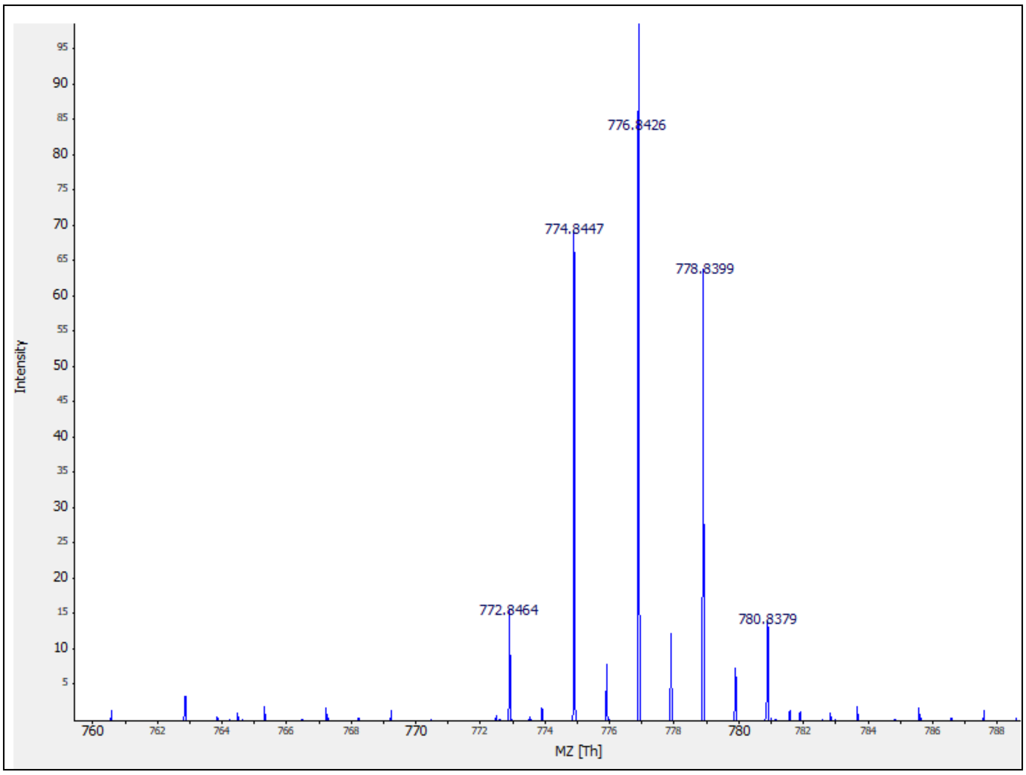

HRESIMS of psammaplysin Z (**1**)

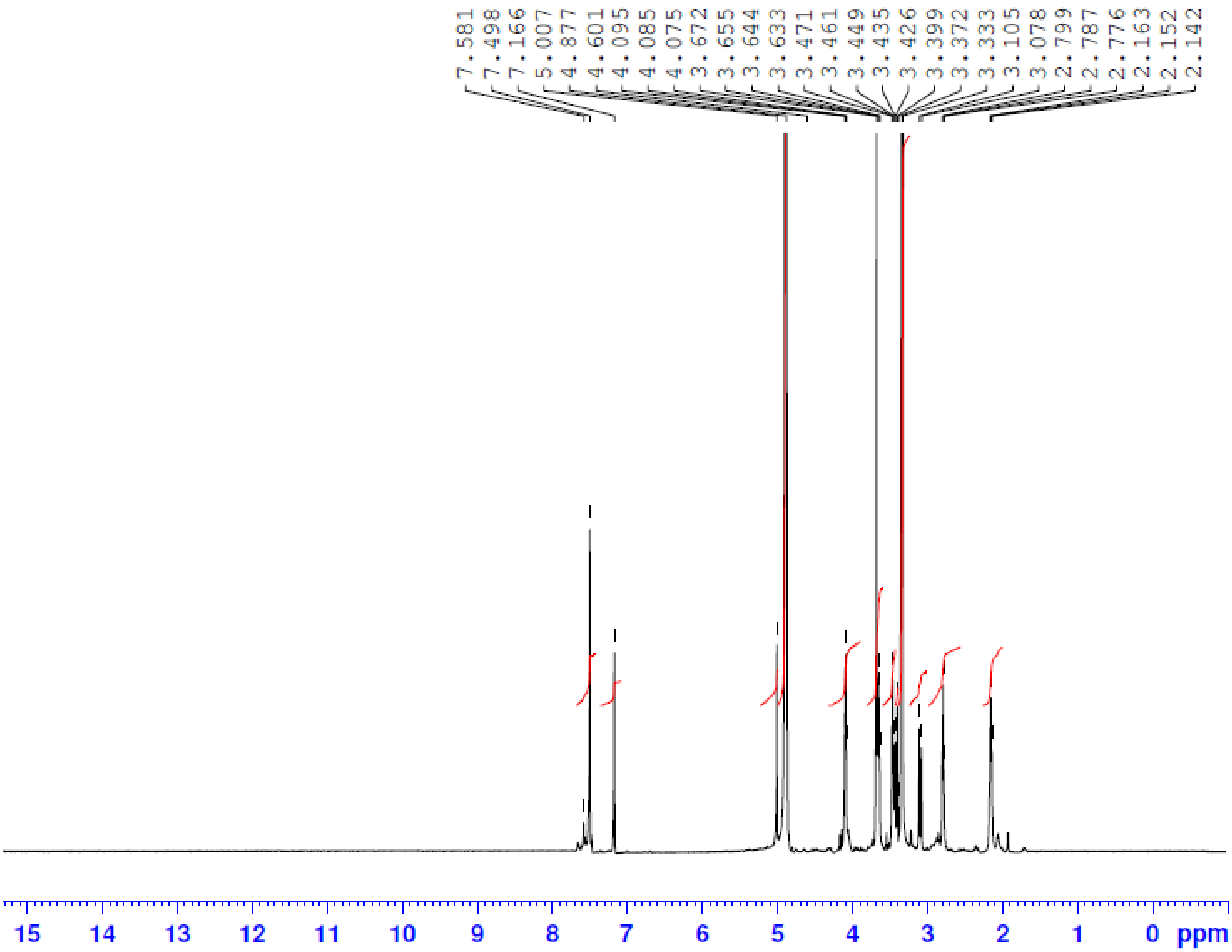

^{1}H NMR spectrum of psammaplysin Z (**1**) (CD_3OD).

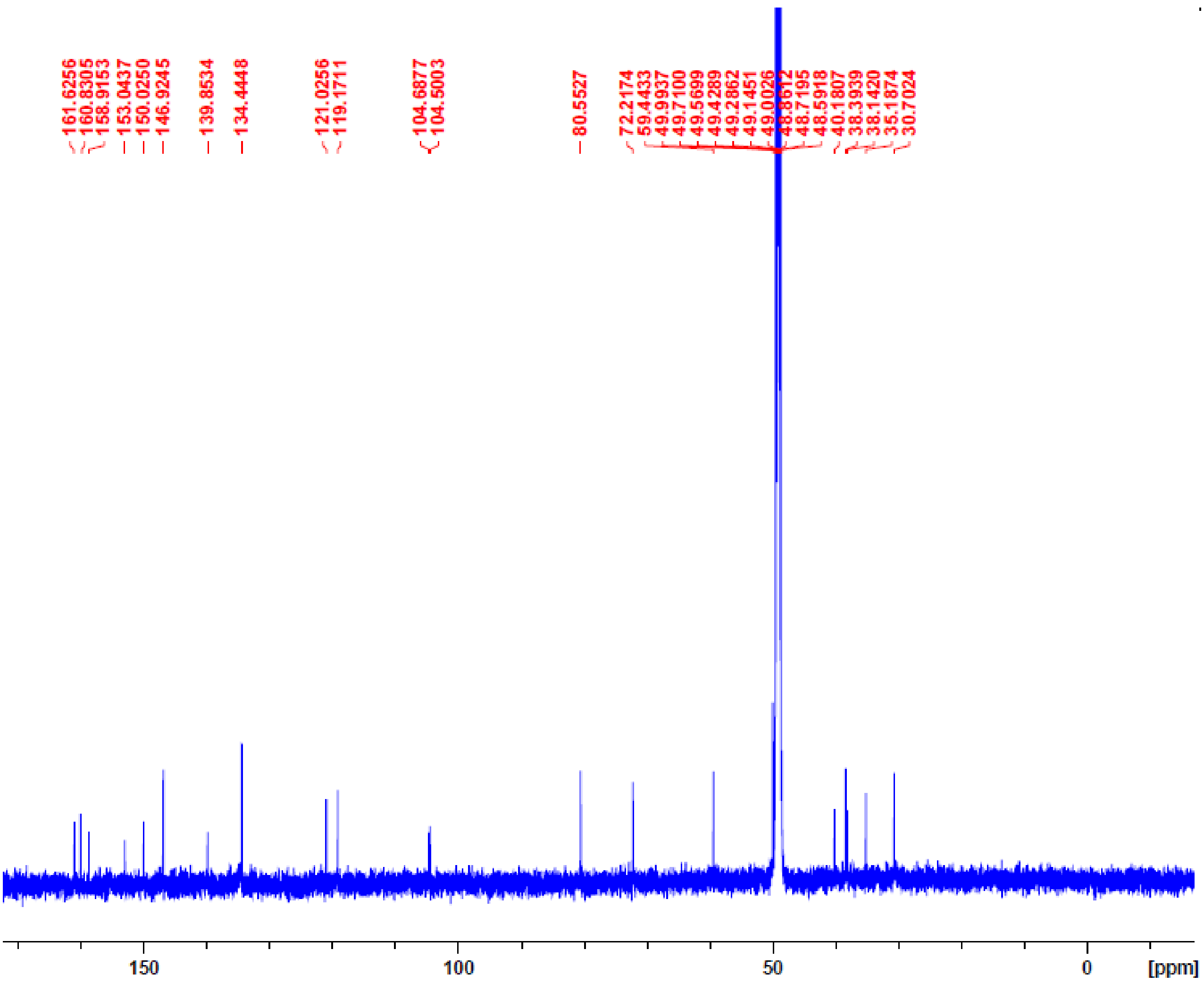

^{13}C NMR spectrum of psammaplysin Z (**1**) (CD_3OD).

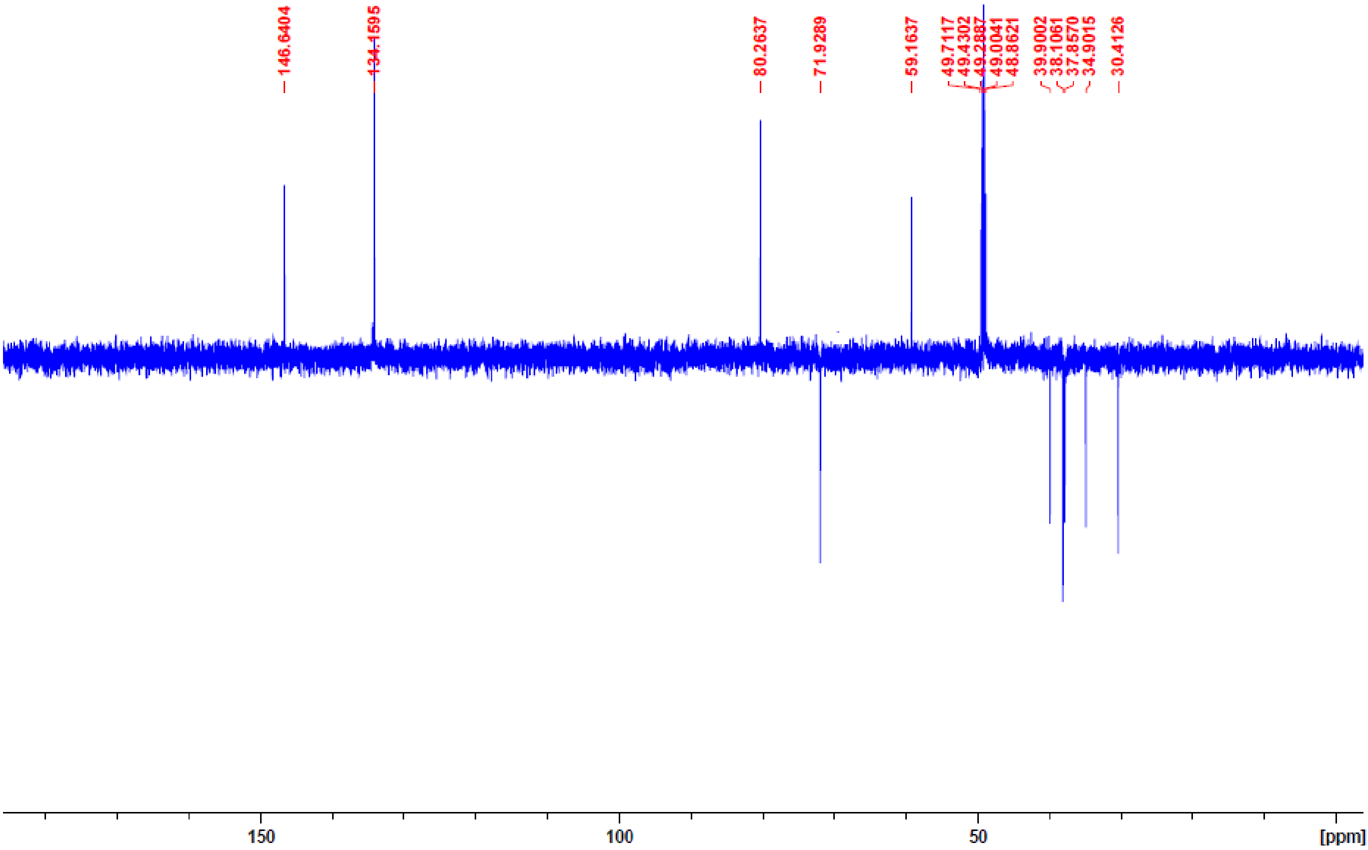

DEPT spectrum of psammaplysin Z (**1**) (CD_3OD).

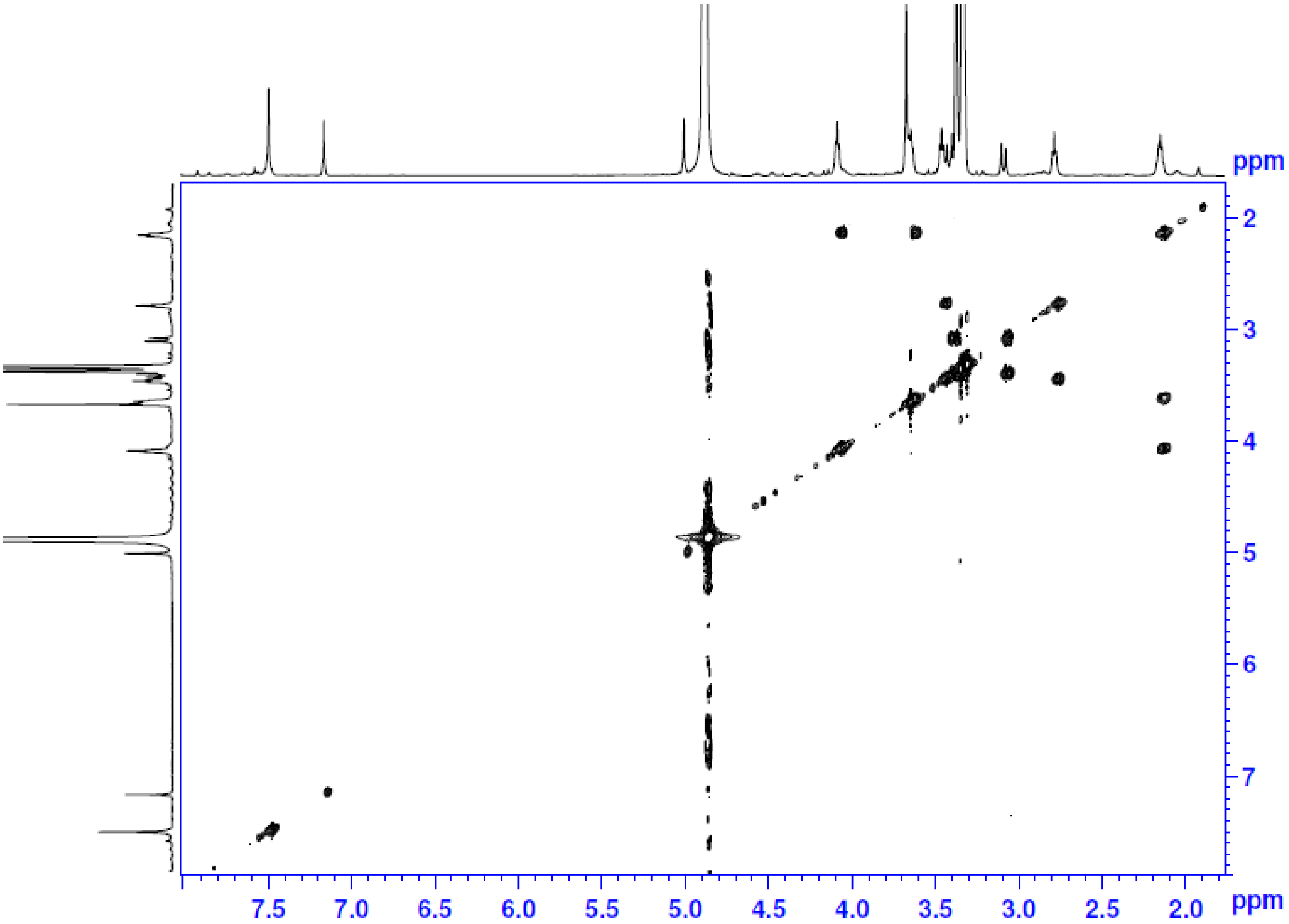

^{1}H-^{1}H COSY spectrum of psammaplysin Z (**1**) (CD_3OD).

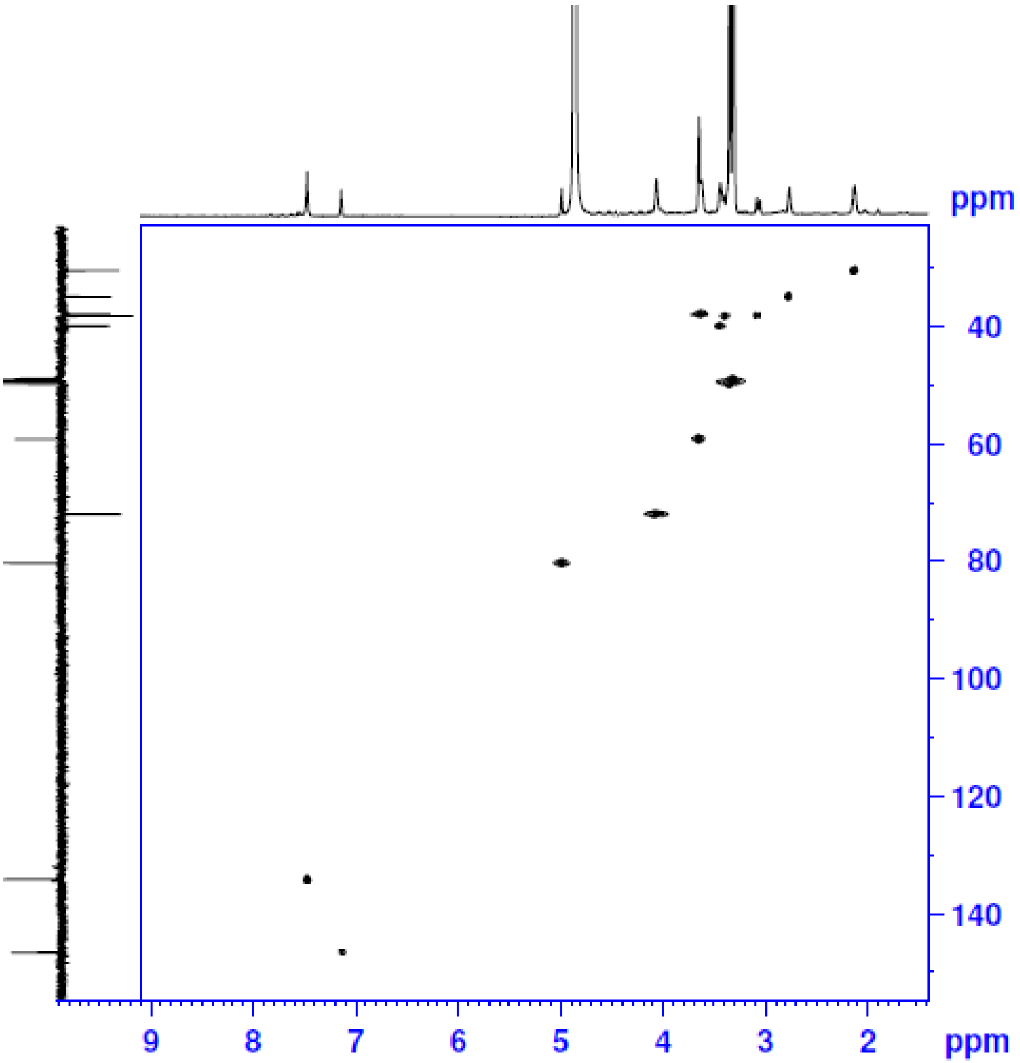

HSQC spectrum of psammaplysin Z (**1**) (CD_3OD)

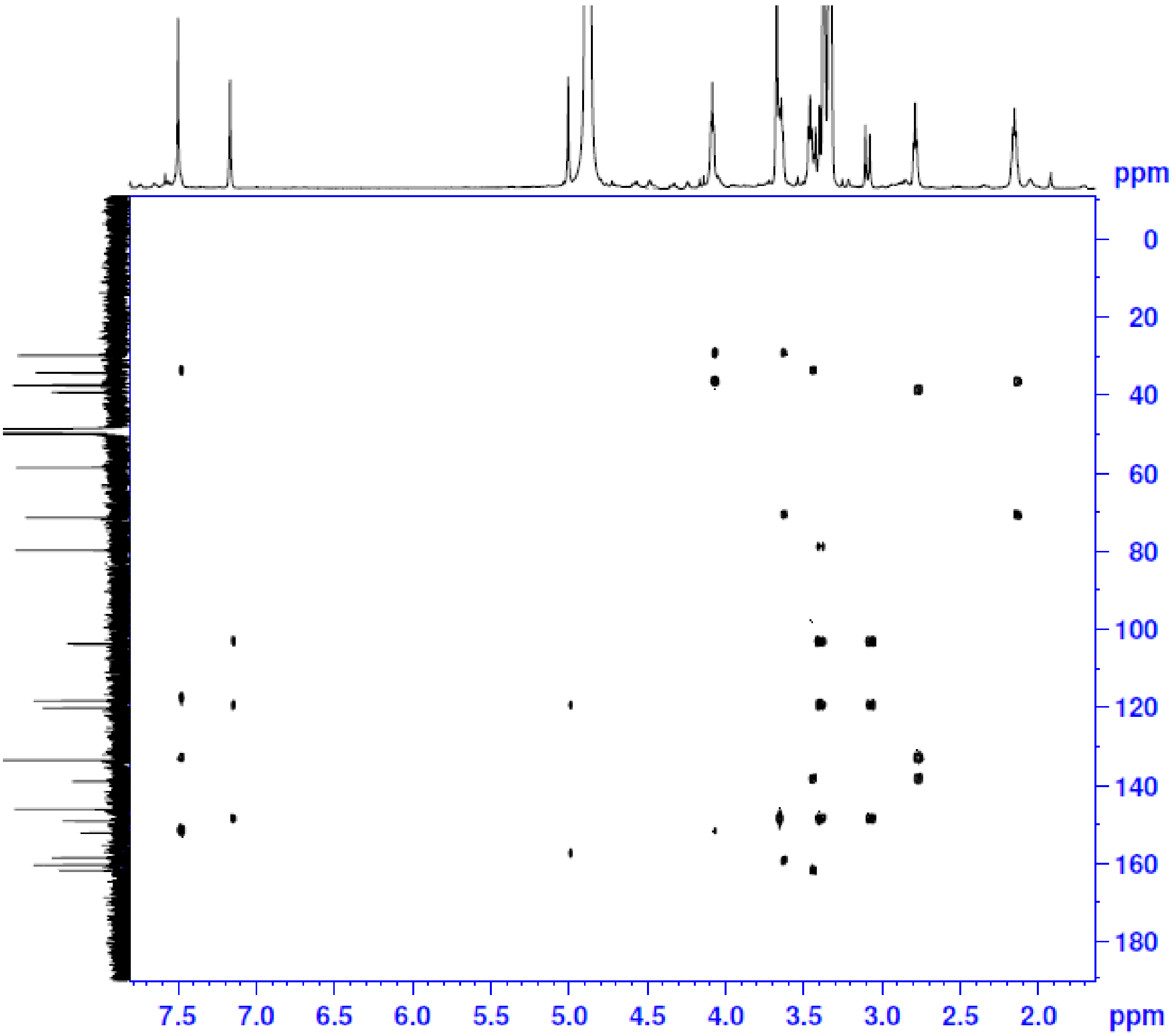

HMBC spectrum of psammaplysin Z (**1**) (CD_3OD)

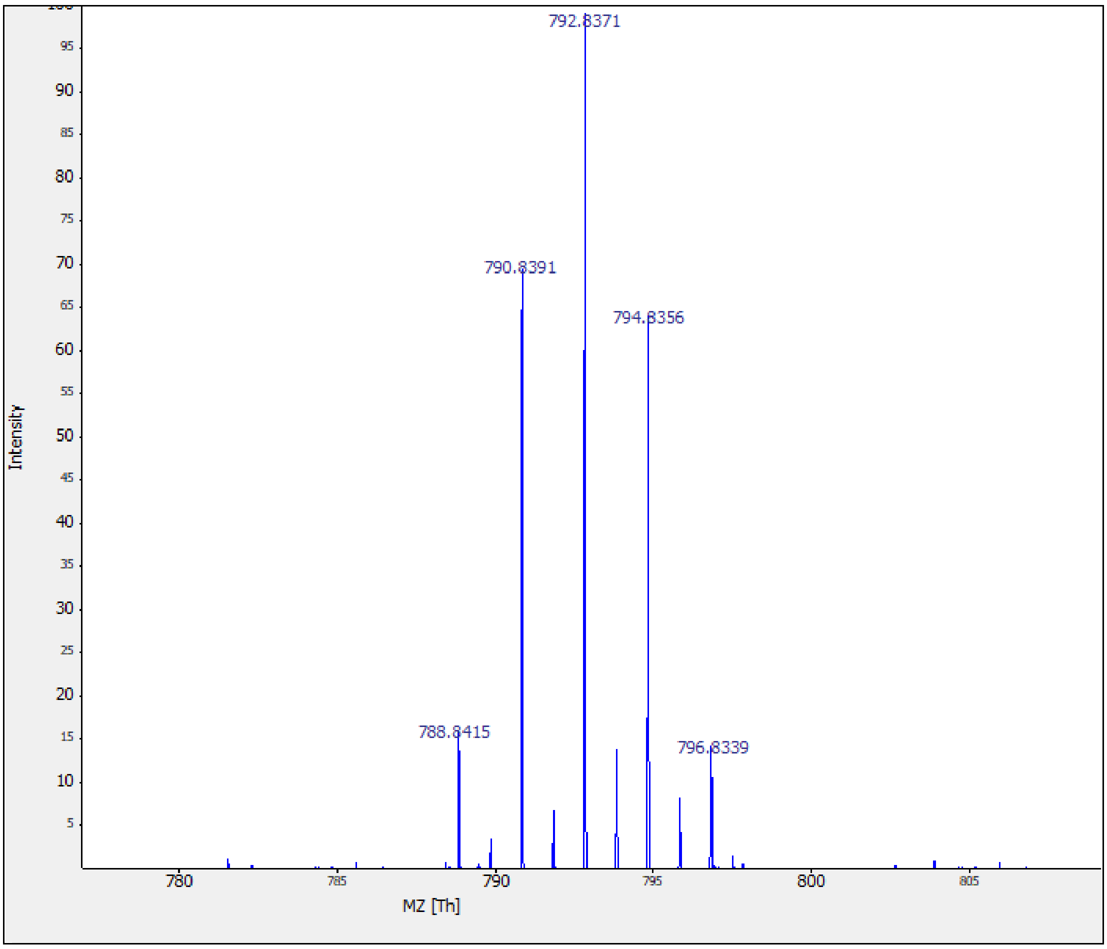

HRESIMS of 19-hydroxypsammaplysin Z (**2**).

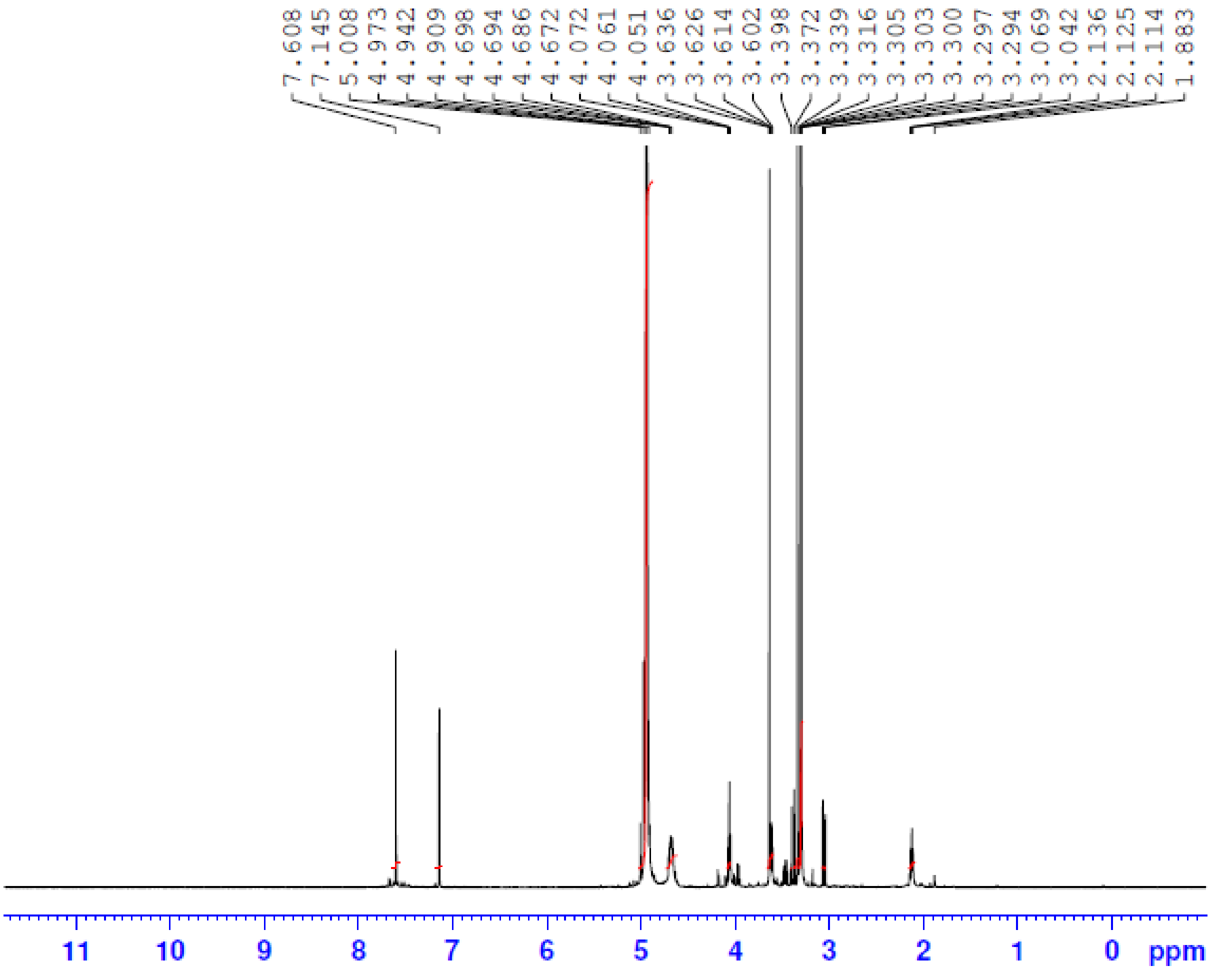

^{1}H NMR spectrum of 19-hydroxypsammaplysin Z (**2**) (CD_3OD).

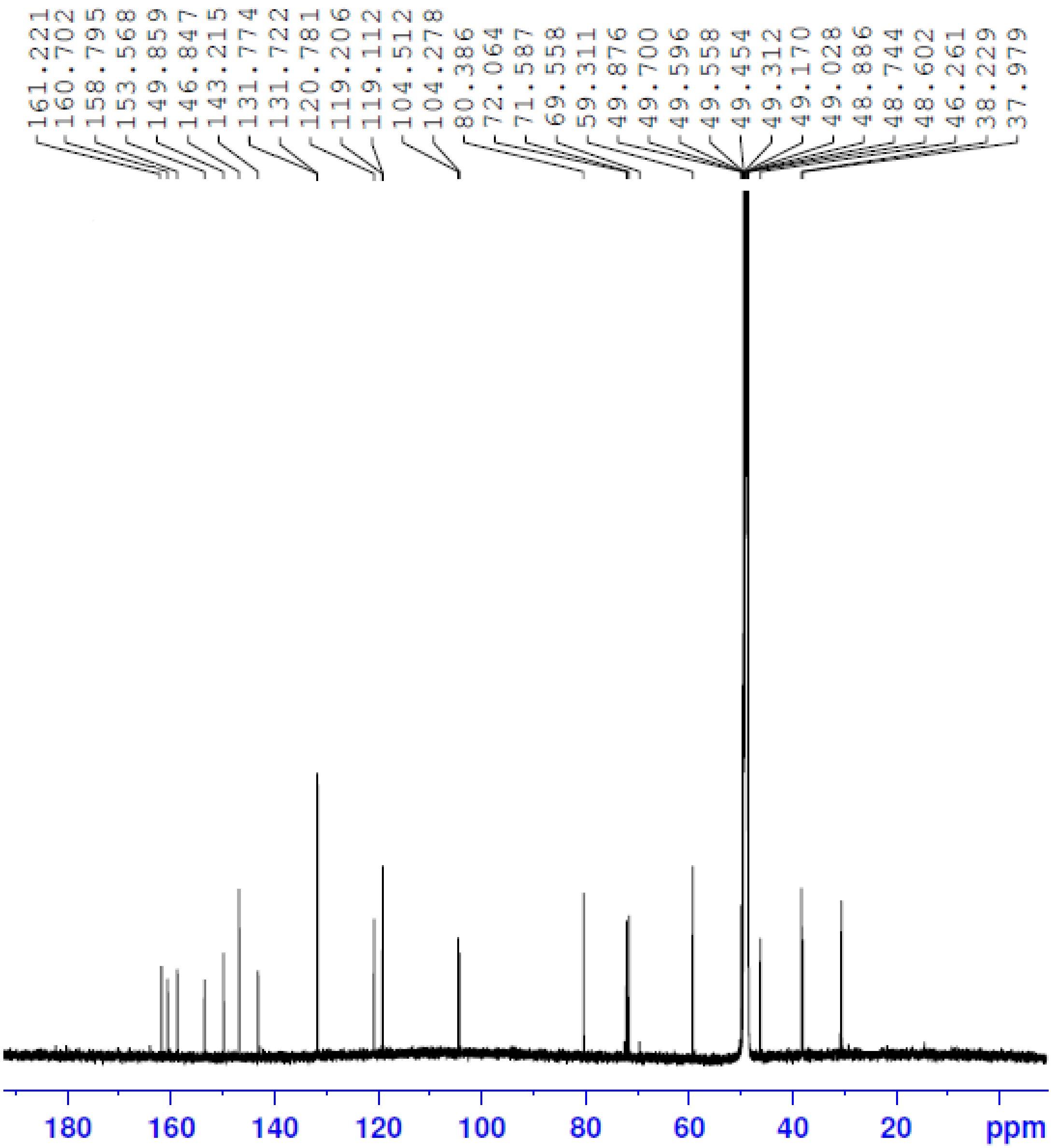

^{13}C NMR spectrum of 19-hydroxypsammaplysin Z (**2**) (CD3OD).

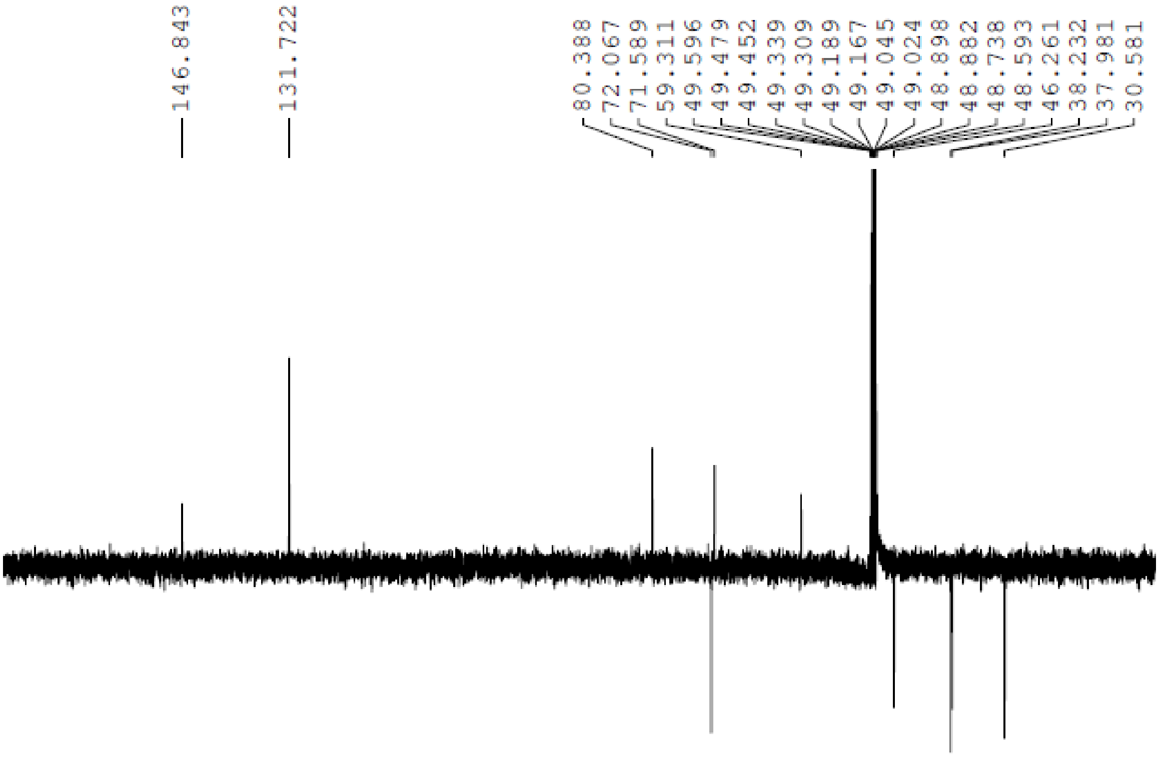

DEPT spectrum of 19-hydroxypsammaplysin Z (**2**) (CD3OD).

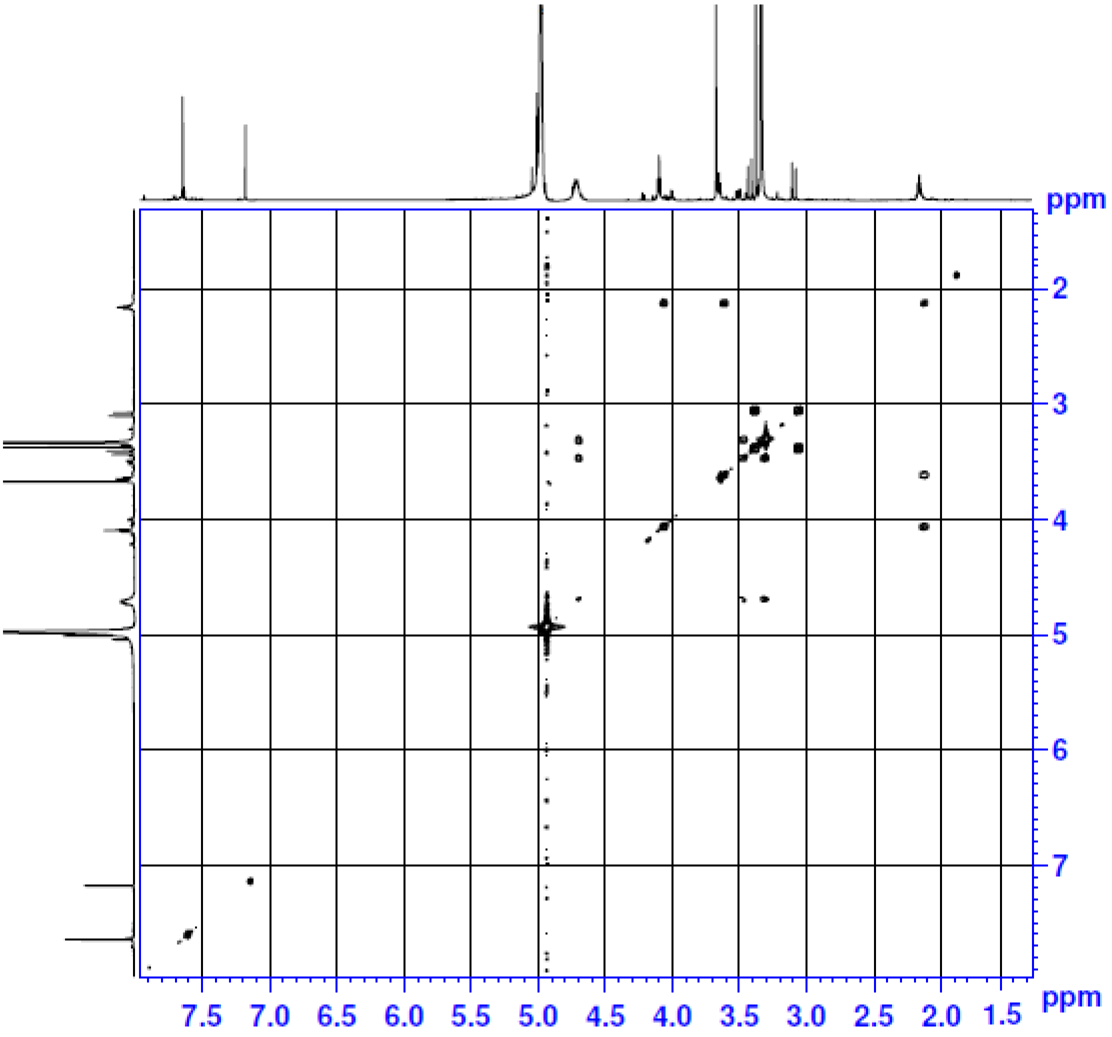

^{1}H-^{1}H COSY spectrum of 19-hydroxypsammaplysin Z (**2**) (CD3OD).

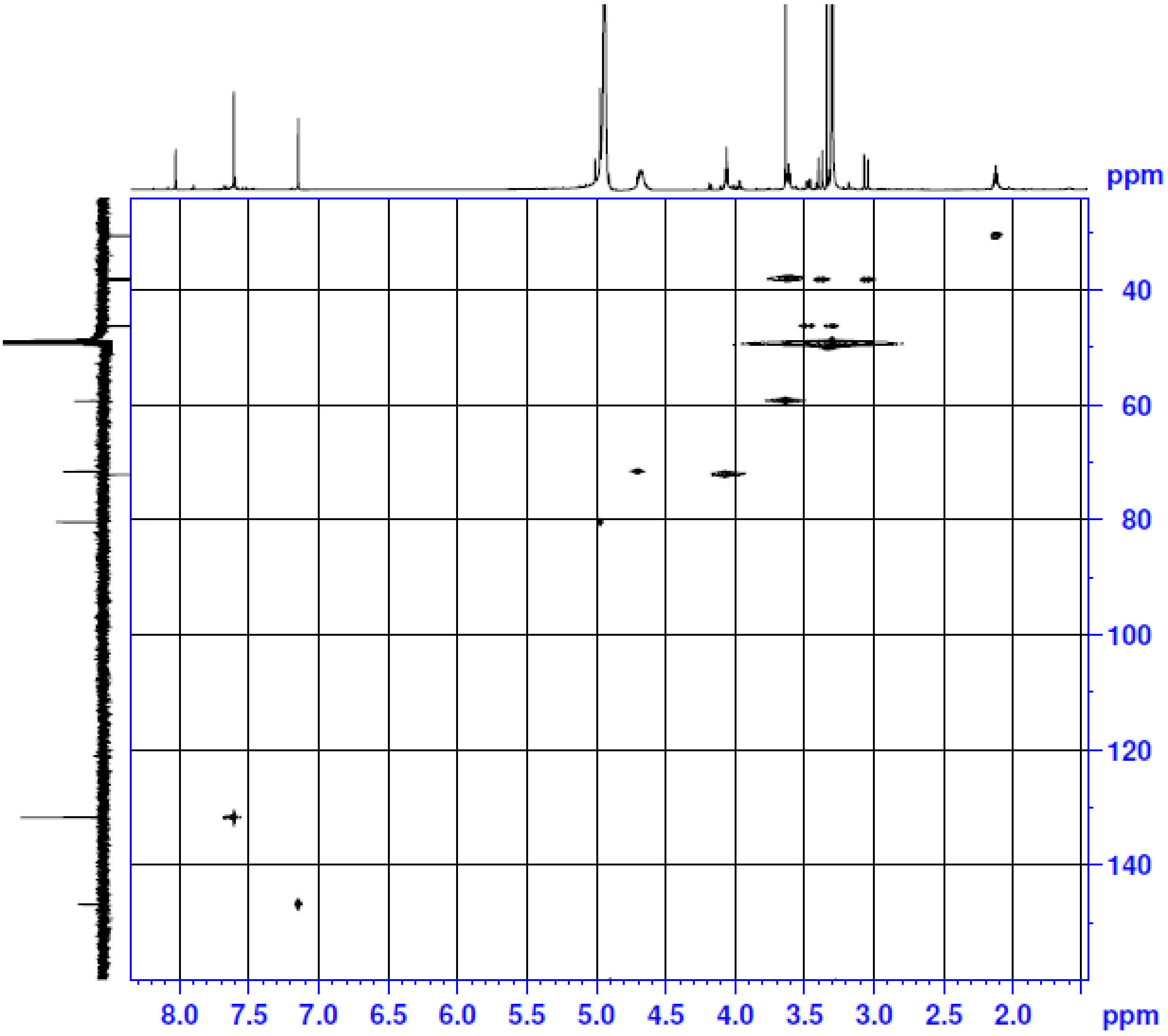

HSQC spectrum of 19-hydroxypsammaplysin Z (**2**) (CD3OD)

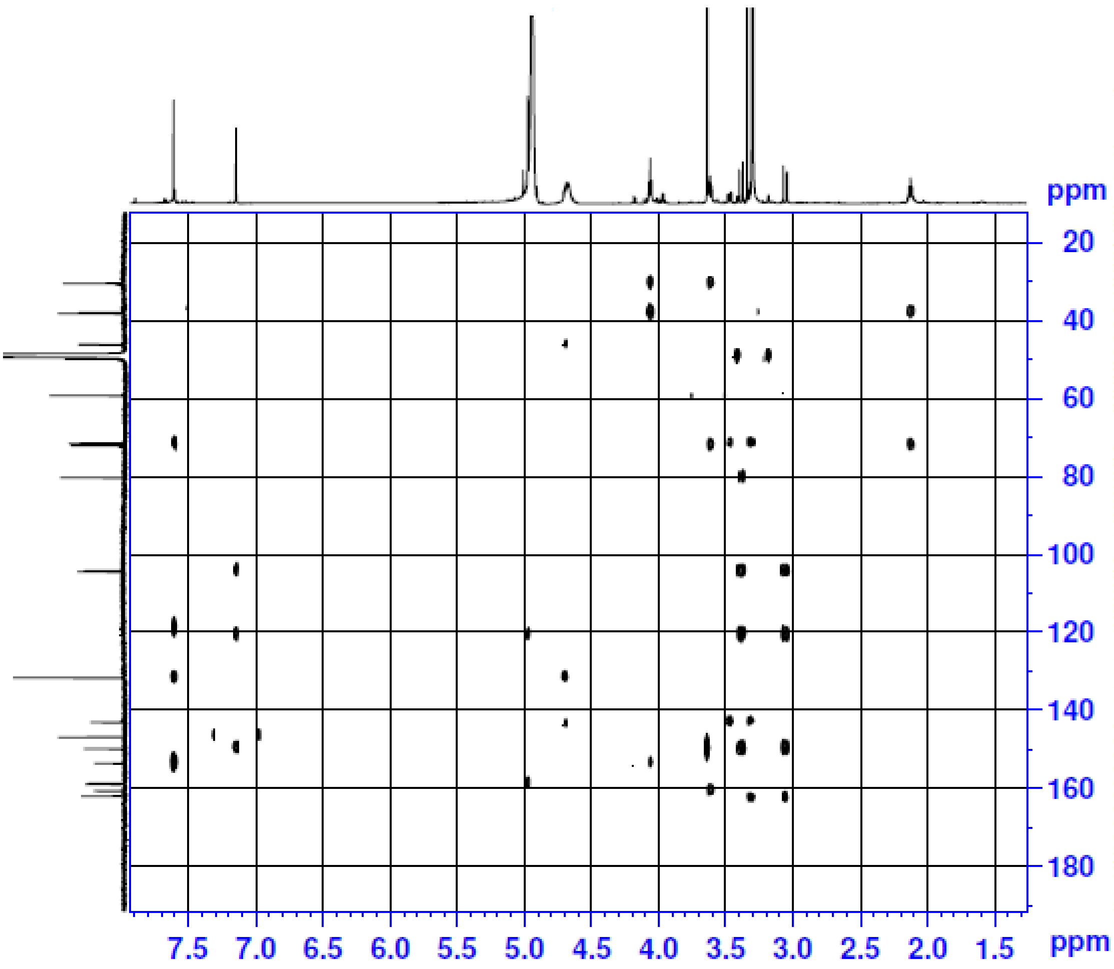

HMBC spectrum of 19-hydroxypsammaplysin Z (**2**) (CD_3OD).
